# Exploring triheptanoin as treatment for short chain enoyl CoA hydratase deficiency

**DOI:** 10.1002/acn3.51359

**Published:** 2021-05-01

**Authors:** Kristin Engelstad, Rachel Salazar, Dorcas Koenigsberger, Erin Stackowtiz, Susan Brodlie, Melanie Brandabur, Darryl C. De Vivo

**Affiliations:** ^1^ Department of Neurology Columbia University Irving Medical Center New York City New York USA; ^2^ Division of Pediatric Gastroenterology, Hepatology and Nutrition New York Presbyterian Hospital New York City New York USA; ^3^ Ultragenyx Pharmaceutical Inc Novato California USA; ^4^ Departments of Neurology and Pediatrics Columbia University Irving Medical Center New York City New York USA

## Abstract

We explored the benefits of triheptanoin as a treatment for Short Chain Enoyl Co‐A Hydratase (SCEH) deficiency. One child with early onset, severe SCEH Deficiency was treated with triheptanoin, an odd chain oil with anapleurotic properties, for 37 months. Blood and urine chemistry safety measures, motor skills assessment, physical exam, and neurological assessment were monitored over a 27 month period. Modest sustained gains in motor skills, attention, muscle bulk, and strength were observed without any significant adverse effects. Triheptanoin appears to be a promising effective treatment for SCEH Deficiency.

## Introduction

Short Chain Enoyl Co‐A Hydratase (SCEH) deficiency is a phenotypically heterogeneous disorder ranging from an early onset severe progressive Leigh‐Like Syndrome with early demise^1^, ^2^ to later onset childhood movement disorders.^3^, ^4^ Early onset symptoms include hypotonia, respiratory insufficiency, global developmental delay, encephalopathy, sensorineural hearing loss, cardiomyopathy, and bilateral basal ganglia lesions.^5^ Less common are the later onset mildly symptomatic patients with varied movement disorders and basal ganglia lesions.^4^, ^5^, ^6^ Intermediate phenotypes also have been reported.^5^, ^7^ Median life expectancy is approximately 2 years.^8^ Last reported age of 24 early onset patients, alive at time of the report, was 104.8 + 95 months (range 8–372 months). Lactate values (blood, CSF, and brain magnetic resonance spectroscopy) in severe cases are elevated, and pyruvate values may be elevated or normal.^5^ In contrast, intermediate and mild cases usually have normal laboratory values. A review of the literature^1^, ^2^, ^3^, ^4^, ^6^, ^7^, ^8^, ^9^, ^10^, ^11^, ^12^, ^13^, ^14^, ^15^, ^16^, ^17^, ^18^, ^19^, ^20^, ^21^, ^22^, ^23^, ^24^, ^25^, ^26^, ^27^, ^28^, ^29^ revealed 67 patients with most suffering the early onset presentation. Average age at death was 28.0 + 43.8 months (range 16 h to 156 months) for 27 early onset patients (age <12 months) for whom age at death was reported.

SCEH deficiency is caused by bi‐allelic mutations in the ECHS1 gene which encodes short chain enoyl CoA hydratase. Mutation types vary with the missense variant being the most common.^23^

**Figure 1:**
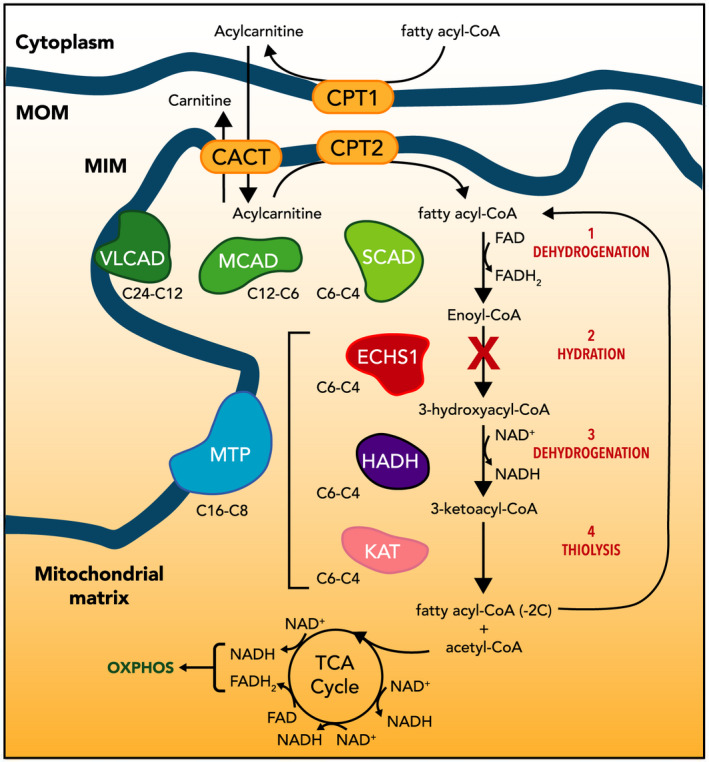
Short chain enoyl Co‐A hydratase deficiency impacts the conversion of enoyl‐CoA to 3‐hydroxyacyl‐CoA in the fatty acid oxidation pathway. 3‐L‐hydroxyacyl‐CoA dehydrogenase (HADH) and 3‐ketoacyl‐CoA thiolase (KAT) complete the conversion to fatty acyl‐CoA.

SCEH is a mitochondrial matrix enzyme that catalyzes the second step of fatty acid beta‐oxidation converting medium and short chain 2‐enoylacyl‐CoA to 3‐hydroxyacyl‐CoA^10^, ^11^, ^18^ (Figure 1), Fatty acid oxidation intermediates are generally normal with some reports of elevated C4 acylcarnitine.^18^ SCEH is also involved in valine metabolism; converting methacrylyl‐CoA to 3‐hydoxybutyryl and acryloyl‐CoA to 3‐hydroxypropionyl‐CoA.^10^ Both methacrylyl‐CoA and acryloyl‐CoA are elevated in patients with SCEH deficiency.^10^, ^30^ Elevated levels of erythro‐2,3 dihydroxy‐2‐methylbutyrate,^7^, ^21^ 3‐methylgluconate, lactate, methylacryloyl‐CoA, and acryloyl‐CoA^21^ also have been reported.

Methacrylyl‐CoA and acryloyl‐CoA toxicity is thought to be the cause of the brain pathology^21^ impairing the pyruvate dehydrogenase complex and the electron transport chain.^18^, ^30^ Pyruvate dehydrogenase deficiency has been reported in the early onset patients but not uniformly.^30^ Sakai et al reported an early onset child, with reductions in respiratory chain complexes, I, III, and IV, but this disturbance has not been found in all patients.

SCEH is also involved with isoleucine and leucine metabolism; however, these metabolites have not been disturbed in patients with SCEH Deficiency, so it is assumed that SCEH does not play a significant role in these pathways.^30^

There is no cure for SCEH Deficiency, but various symptomatic treatments have been attempted. The ketogenic diet has generally not been helpful, although there is one mild patient whose dystonia improved.^4^ Vitamin cocktails are not useful. Dystonia in one mild patient improved on a low protein diet.^4^ Another mild patient was given carnitine, creatine, and idebenone without effect.^4^ Levodopa was evaluated for movement disorders without effect.^4^ Two patients on a low valine diet and a vitamin cocktail showed motor skill improvements.^26^ Three patients on a low valine diet showed improvements in awareness, muscle tone, spontaneous movement, and language.^31^

Triheptanoin (C7 oil) has been used to treat various fatty acid oxidation disorders^32^, ^33^, ^34^, ^35^ and Glut‐1 Deficiency Syndrome^33^, ^36^ with minimal to significant improvement in clinical symptoms. In most studies, triheptanoin replaced 30%–40% of daily calories^32^, ^36^ in an otherwise normal diet. The C7 oil is well‐tolerated; however, GI symptoms have been reported.^36^ Triheptanoin replenishes the TCA cycle intermediates (known as anaplerosis), gluconeogenesis, and seems not to depend on SCEH for metabolism. Therefore, a trial of C7 oil appeared warranted in patients with SCEH deficiency.

**Figure 2:**
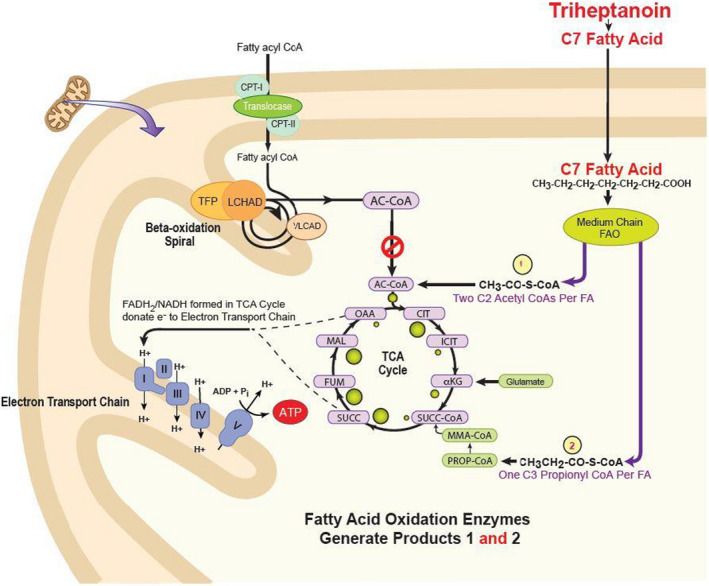
Triheptanoin enters the mitochondria as a C7 fatty acid, each of which is metabolized to two C2 Acetyl CoA's, which directly enter the TCA cycle, and one C3 Propionyl CoA that is converted to methylmalonyl‐CoA and then to succinyl‐CoA, a TCA intermediate.

Triheptanoin is a naturally occurring medium odd‐chain triglyceride which directly enters the mitochondria without the need for carnitine.^37^ In the liver, C7 oil is metabolized to four carbon (acetoacetate and betahydroxybutyrate) and five carbon ketone bodies (Beta‐hydroxypentanoate (BHP) and Beta‐ketopentoate (BKP)) that are exported to peripheral tissues, including the brain, where they are converted to acetyl‐CoA and propionyl‐CoA.^32^ Propionyl CoA is then converted to succinyl‐CoA (by propionyl CoA carboxylase and methylmalonyl CoA mutase) before entering the TCA cycle directly (Figure 2). Propionyl‐CoA also contributes to gluconeogenesis. Triheptanoin enters the mitochondria largely as the carboxylate, without the need for CPTI, carnitine‐acylcarnitine translocase or CPTII.^37^ It is then metabolized sequentially to acetyl CoA by medium chain‐acyl‐CoA synthetase, MCAD, IVCD, SCAD, and thiolase.

## Methods

A female child with SCEH deficiency was treated with daily dosing of triheptanoin after approval of a single patient Investigational New Drug (IND) application. Ultragenyx Pharmaceuticals provided the triheptanoin which was administered enterally on an ascending dose regimen (5% to 40% of total daily calories divided into seven daily doses). Safety measures (blood and urine chemistries, EKG), neurological examination, physical examination, and motor function assessments were performed quarterly. Outcome measures included the following: (1) The Columbia Neurological Score (CNS),^38^ which includes a several domain physical and neurological exam, with normal range (70–76), mild impairment (60–69), moderate (50–59), and severe (40–49); (2) The Gross Motor Function Measure‐88 (GMFM‐88) which measures motor changes in neuromuscular disorders^39^, ^40^ (minimum score is 0 and maximum is 264); and (3) The Children's Hospital of Philadelphia Infant Test of Neuromuscular Disorders (CHOP‐INTEND) which measures motor function for fragile infants with neuromuscular disorders (minimum score is 0 and maximum is 64).^41^

## Results

The patient, a 6 year 11 month old girl, presented at age 6 months with Leigh syndrome, global developmental delay, ptosis, oscillatory eye movements, mixed tone abnormalities, hyperreflexia, and bilateral Babinski signs. Compound heterozygous mutations in the ECHS1 gene (p. Ala173Val and p. Gly175Ser), confirmed the diagnosis of SCEH deficiency. Brain MRI at age 8 months showed mildly prominent CSF spaces, moderate overall symmetrical diminution in cerebral white matter volume, subtle nonspecific symmetric restricted diffusion, and T2 prolongation within the basal ganglia, especially posteriorly and inferiorly, and including the cerebral peduncles/subthalamic regions. Plasma acylcarnitine values were normal and urine organic acids showed elevated 3‐methylglutaconic acid values.

**Figure 3:**
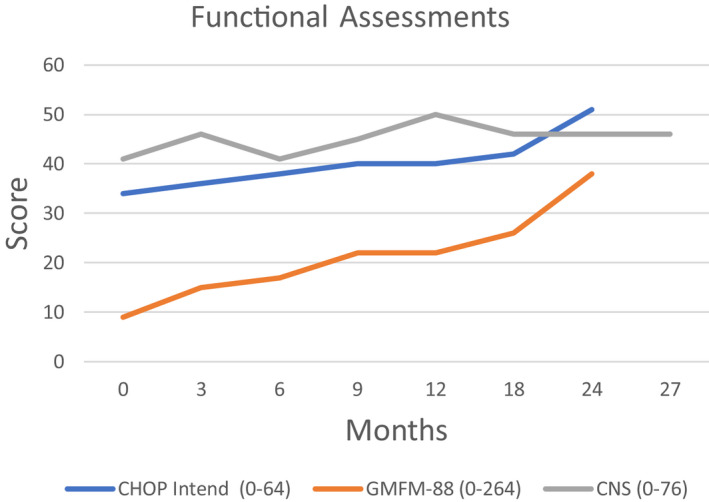
Repeat assessment of functional ability was obtained over a 27 month period using the CHOP Intend, GMFM‐88 and the CNS, all widely used measures of motor skill.

At age 47 months, treatment with triheptanoin was initiated and has continued through age 6 years and 11 months. C7 oil was administered orally as an ascending dose (5% to 40% of total daily calories divided into 7 daily doses). The patient's condition has remained stable clinically since starting the C7 oil with subtle improvements in awareness, posture, purposeful movements, affect, and sleep pattern. The CHOP‐INTEND Score improved 17 points during the first 24 months of treatment (baseline score 34/64; 24 month score 51/64), and the GMFM‐88 improved 29 points (baseline score 9/264; 24 month score 38/ 264) (Figure 3). The CNS score improved 5 points (baseline score 41/76; 27 month score 46/76) reflecting increases in muscle bulk and strength. Acylcarnitine values showed increases from baseline to 27 months of treatment: (1) Acetyl [C2] 10.9 to 25.02 umol/L, (2) Propionyl [C3] 1.13 to 5.32 umol/L, and (3) Isovaleryl/2‐methlybutyryl [C5] 0.31 to 0.88 umol/L (Table 1). Urine organic acids levels varied. Lipid and hepatic profiles, basic metabolic panel, and complete blood count values were normal throughout. There has been no evidence of clinical regression or serious side effects over the 37 months of treatment. In‐person evaluations were interrupted after 27 months due to COVID‐19 restrictions.

**Table 1:** Selected blood and urine chemistries.

Chemistry (normal range)	BL	3 months	6 months	9 months	12 months	18 months	24 months	27 months
Plasma acylcarnitine profile (normal range)								
Acetyl [C2] (3.69–24.71 µmol/L)	10.9	15.2	14.5	29.24	12.3	19.2	39.01	25.02
Propionyl [C3] (≤0.97µmol/L)	1.13	4.28	4.9	8.65	3.2	7.18	8.36	5.32
Isobutyryl [C4] (≤0.50 µmol/L)	0.99	0.85	0.7	0.63	0.7	0.68	0.76	0.67
Isovaleryl/2–Methylbutyryl [C5] (≤0.28 µmol/L)	0.31	0.44	0.6	1.39	0.6	1.0	1.43	0.88
Urine organic acids (nmols)								
3–methylglutaconic acid (≤10 nmols)	present	present	present	present	ND	36	present	ND
Methylmalonic Acid (0–5 nmols)	3	6	5	9	ND	6	2	ND
3–OH Butyric Acid (0–4 nmols)	4	5	6	142	ND	344	8	ND
Acetoacetic Acid (0–4 nmols)	0	0	0	58	ND	246	0	ND
Adipic Acid (0–35 nmols)	36	16	19	51	ND	12	34	ND
Pyruvic Acid (0–30 nmols)	59	47	24	25	ND	4	16	ND
Plasma lipid profile								
HDL (40–60mg/dl)	30	33	29	33	31	34	34	29
Hepatic function panel	nl	nl	nl	nl	nl	nl	nl	ND
Complete blood count	nl	nl	nl	nl	nl	nl	nl	ND
Basic metabolic panel								
Creatinine (0.4–0.70 mg/dl)	0.29	0.34	0.29	0.48	0.31	0.3	ND	ND
AGAP (5–17)	19	18	15	11	18	15	ND	ND

## Discussion

We present a child with severe, early onset SCEH Deficiency treated successfully with triheptanoin. The patient presented early in infancy with clinical signs and laboratory findings typical of SCEH deficiency including severe developmental delay, abnormal brain MRI, elevation of urinary 3‐methylglutaconic acid, and bi‐allelic mutations in the ECHS1 gene. As expected, treatment with C7 oil resulted in increased medium and short chain acylcarnitine levels and elevations of urinary ketones. Baseline motor performance was severely delayed but stabilized after starting triheptanoin, followed by mild improvements in motor performance, head control, and sitting tolerance during the first 12 months. Motor skills continued to improve thereafter. Of note, there has been no clinical worsening which is commonly seen in untreated infantile onset disease. We were concerned when starting Triheptanoin as to whether SCEH is important in the metabolism of this heptanoate or any of its metabolites. The clinical improvement and the lack of any toxicity suggests that SCEH is not necessary or critical in the metabolism of these metabolites. Lifespan, at this point, has exceeded that of most early onset patients with SCEH deficiency. We have attributed the improvements in overall energy level, to C7 oil which replenishes TCA cycle intermediates, supports gluconeogenesis, and replaces fats commonly found in a standard diet. Daily total calories were maintained on a strict regimen accommodating standard protein and carbohydrate recommendations, and triheptanoin accounted for 40% of total daily calories. Limited protein intake accounted for reductions in dietary valine, but it is unclear whether decreased valine intake was therapeutic. Since most patients have the early onset severe phenotype, initiating treatment in the newborn period would seem to be ideal. Newborn screening would facilitate the diagnosis of genetically affected infants before or soon after onset of clinical symptoms permitting early treatment and protection of the developing nervous system. One of the limitations of this study is small sample size and delayed treatment. Continued evaluation of triheptanoin as a promising effective treatment for SCEH Deficiency is an important next step.

## Conflict Of Interest

Melanie Brandabur is paid by Ultragenyx Pharmaceutical Inc.
